# Pulmonary Coccidioidomycosis: A Case Report and Literature Review

**DOI:** 10.3390/medicina58050655

**Published:** 2022-05-12

**Authors:** Tadas Alčauskas, Birutė Zablockienė, Rolandas Zablockis, Linas Svetikas, Laura Bilotaitė, Ligita Jančorienė

**Affiliations:** 1 Vilnius University Faculty of Medicine, Vilnius University, M.K. Ciurlionio 21, LT-03101 Vilnius, Lithuania; laura.bilotaite@mf.stud.vu.lt; 2 Clinic of Infectious Diseases and Dermatovenerology, Institute of Clinical Medicine, Vilnius University Faulty of Medicine, Vilnius University, M.K. Ciurlionio 21, LT-03101 Vilnius, Lithuania; birute.zablockiene@santa.lt (B.Z.); linas.svetikas@santa.lt (L.S.); ligita.jancoriene@santa.lt (L.J.); 3 Clinic of Chest Diseases, Immunology and Allergology, Faculty of Medicine, Institute of Clinical Medicine, Vilnius University, M.K. Ciurlionio 21, LT-03101 Vilnius, Lithuania; rolandas.zablockis@santa.lt

**Keywords:** coccidioidomycosis, fluconazole, fungal infection

## Abstract

Coccidioidomycosis is an infectious disease caused by *Coccidioides immitis* or *C. posadasii* fungus. Humans usually get infected by inhaling spores risen from the soil. Although in 60 percent of cases symptoms are absent, remaining patients can develop various manifestations of the disease, from flu-like symptoms to severe dissemination or meningitis. In endemic regions (California, Arizona, Mexico, Central, and South America), pulmonary coccidioidomycosis causes 25% of community-acquired cases of pneumonia. We present the first registered case of pulmonary coccidioidomycosis in Lithuania. Clinical presentation, pathogenesis, treatment options, and diagnostic alternatives are discussed.

## 1. Introduction

Coccidioidomycosis is an infectious disease caused by *Coccidioides immitis* or *C. posadasii* fungus. The disease is endemic in California, Arizona, Mexico, Central, and South America [1,2]. Most cases in the United States of America are registered in Arizona (about two-thirds), and California is second-highest. The highest incidence in California is registered in these central counties: Fresno, Kern, Kings, San Luis Obispo, and Tulare [3]. Even though in 60% of cases the symptoms are absent, the remaining patients can develop various manifestations of the disease: from flu-like symptoms to severe dissemination or meningitis [4].

Fungus of the *Coccidioides* genus expresses dimorphism and alternation between saprophytic (micellar) and spherical (infective) phases. Therefore, humans usually get infected by inhaling arthroconidia spores risen from the soil [5,6]. It is unclear if inhaled spores can enter the alveoli. It is thought that spores mainly damage terminal and respiratory bronchioles as the size of spores usually ranges from 3 to 5 μm [5,7]. Individual susceptibility to the infection depends on various biosocial factors, such as age, gender, work conditions, comorbidities, and immunosuppression [1,8]. Even though Lithuania is not an endemic coccidioidomycosis region, more and more residents are traveling to the endemic countries, and the risk of imported disease is getting higher [9]. We present the first recorded case of coccidioidomycosis in Lithuania, which was imported from California.

## 2. Case Report

The 31-year-old male went to a family doctor because of a persistent cough and general weakness lasting for one week. He had neither chronic comorbidities nor allergies, denied use of any drugs or harmful habits, such as excessive usage of alcohol or smoking. The patient was touring Sequoia National Forest in Tulare County, California, before his illness, where he spent three weeks tenting in the wilderness. Chest X-rays showed an infiltration in the lower segments of the right lung, and amoxicillin and clavulanic acid was prescribed. After two days, the patient developed a fever of 38 °C and again consulted a family doctor. Blood analysis showed leukocytosis: white blood cell (WBC)–17.0 × 10^9^/L (normal range: 4.0–9.8 × 10^9^/L); eosinophils–6.0 × 10^9^/ L (normal range: 0.0–0.7 × 10^9^/L); neutrophils 7.8 × 10^9^/L (normal range: 1.5–6.0 × 10^9^/L); and elevated C-reactive protein (CRP)–89.9 mg/L (normal range: 0.0–5.0 mg/L). The patient was hospitalized in Vilnius University Hospital Santaros Clinics (VUH SC) for further examination. 

**Figure 1:**
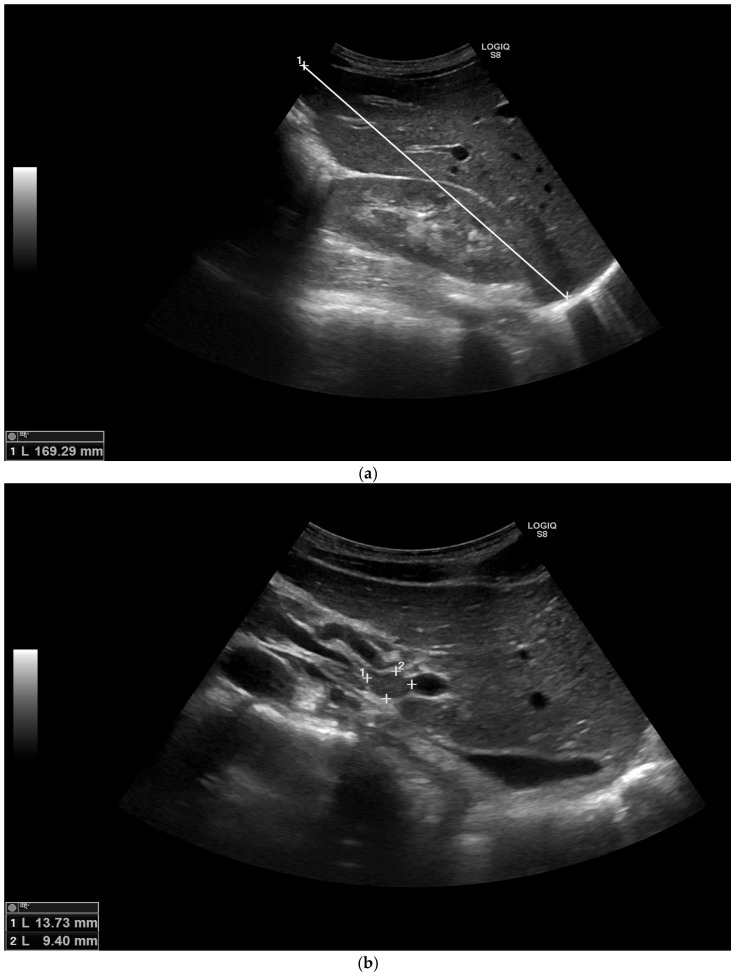
(**a**) Ultrasound of the abdomen showing hepatomegaly: liver diameter is 169.29 mm in the midclavicular line (normal range < 150.00 mm). (**b**) Ultrasound of the abdomen showing portal hepatic lymphadenopathy: lymph node dimensions 13.73 × 9.40 mm.

**Figure 2:**
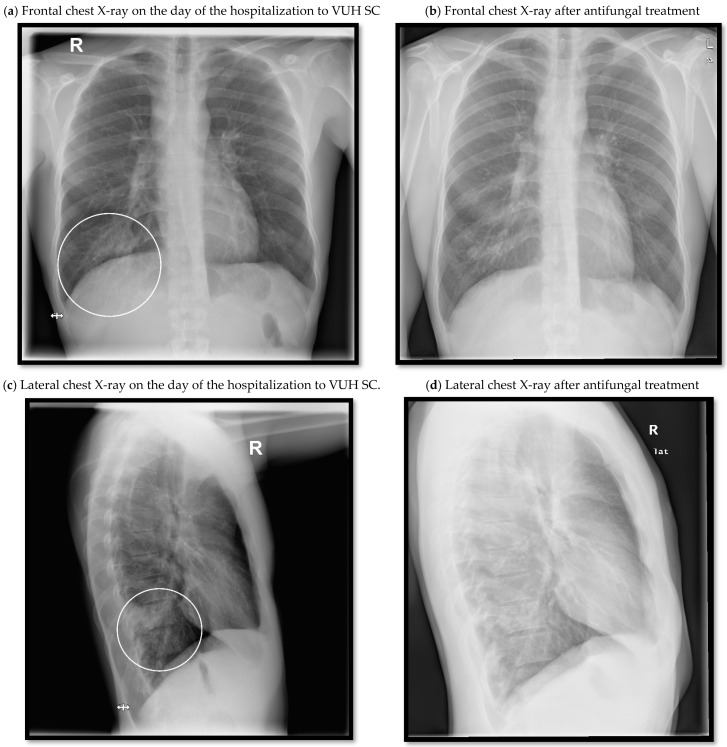
Frontal and lateral chest X-rays showing dynamics of the infiltration in the basal segment of the right lung.

Additional laboratory analysis revealed elevated liver enzymes: serum glutamic pyruvic transaminase (GPT)–133.0 U/L (normal range: ≤40.0 U/L), serum glutamic oxaloacetic transaminase (GOT)–71.0 U/L (normal range: ≤40.0 U/L). Hepatomegaly, portal hepatic lymphadenopathy and gallbladder polyps were seen in the ultrasound of the abdomen (Figure 1a,b). The chest X-ray was repeated (Figure 2). Differential diagnosis was made between eosinophilic granulomatosis with polyangiitis (EGPA) and parasitosis. However, EGPA was rejected because the patient did not have any signs of renal damage or bleeding into the alveolar spaces of the lungs. 

**Figure 3:**
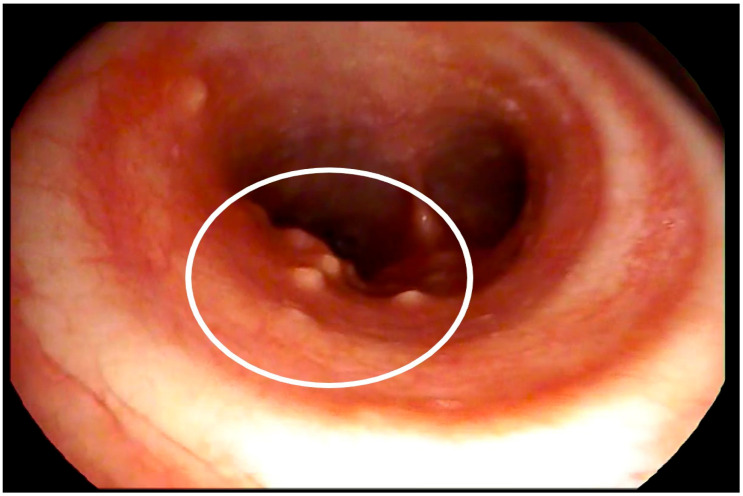
Fibrobronchoscopy showing multiple nodules in the trachea.

Because of pulmonary damage and evident eosinophilia, a bone marrow trephine biopsy was carried out. No malignant haematological changes were found. Bronchoscopy, endobronchial biopsy, transbronchial lung biopsy, and endobronchial ultrasound-guided (EBUS) mediastinal lymph node biopsy did not reveal any malignant cells; however, multiple nodules were seen in the trachea during bronchoscopy (Figure 3). Histological analysis revealed massive monomorphonuclear and eosinophilic infiltration in trachea nodules and dominant areas of lymphocytes with an abundant mixture of eosinophils and neutrophils in the mediastinal lymph node. Multinucleated giant cells, collections of epithelioid histiocytes, and a couple of necrotic areas were also observed. Laboratory investigations did not show acid-resistant bacteria nor Mycobacterium tuberculosis complex DNA. Serum markers of autoimmune diseases were also negative. 

Laboratory serum analysis for paragonimiasis, schistosomiasis and strongyloidiasis came negative. During hospitalization, the patient was treated with imipenem/cilastatin 500/500 mg q6hr intravenously and doxycycline 100 mg q12hr perorally. The patient’s condition stabilized, blood inflammatory indicators decreased, febrile fever disappeared, and infiltration seen in the chest X-ray reduced. The patient was discharged for outpatient treatment under the supervision of a pulmonologist. 

**Figure 4:**
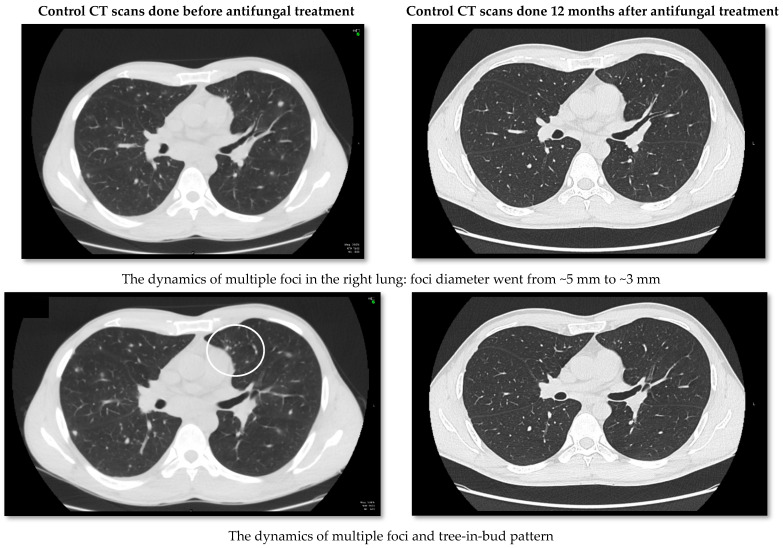

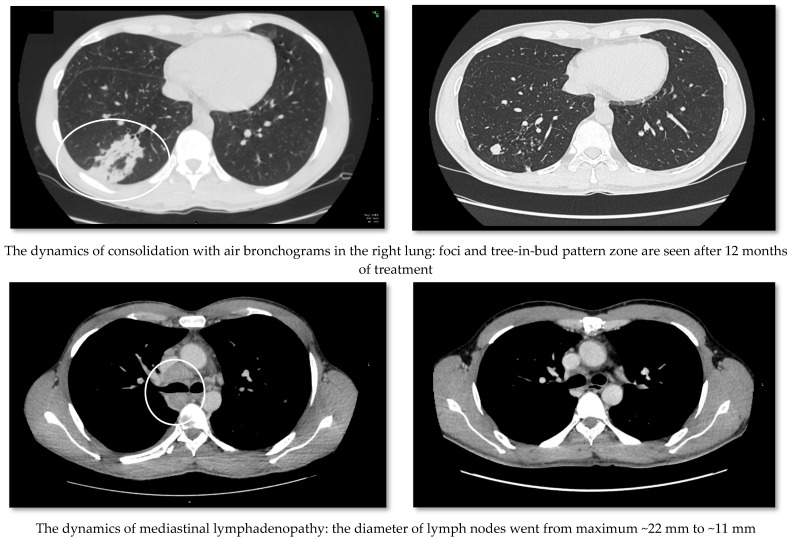
The dynamics of control CT scans before treatment and after 12 months of treatment.

Several computerized tomography scans (CT) were made to monitor the damage to the lungs. Three months after the onset of the symptoms, mediastinal lymphadenopathy and tree-in-bud pattern zone with focal lung lesions in S9/10 segments of the right lung remained (Figure 4). Eosinophilia in peripheral blood also was persistent (1.12 × 10^9^/L). Four months after the disease manifestation, the results of a sputum culture came from the referential laboratory at the Robert Koch Institute, Germany, and the growth of *Coccidioides immitis* was observed. 

After confirmation of the coccidioidomycosis diagnosis, the patient was prescribed fluconazole 400 mg a day perorally. After 12 months of treatment, the patient had no respiratory symptoms, eosinophilia disappeared, the tree-in-bud focal lesions of the S9/10 segments and mediastinal lymphadenopathy seen on the CT scan got smaller (Figure 4), and treatment was terminated. To monitor possible signs of renewal of the infection, the patient undergoes CT scans every 6 months.

## 3. Literature Review

### 3.1. Clinical Features and Diagnostics

Pulmonary coccidioidomycosis causes 25 percent of community-acquired pneumonia in endemic regions [10]. It takes 1 to 3 weeks for primary symptoms to develop after the spores enter the body. One of the first immune response signs is the development of erythema nodosum or erythema multiforme on the patient’s skin [11]. Segmental or lobar consolidations and mediastinal lymphadenopathy are incident in the chest X-ray [10]. It is noticed that in from 5 to 15 percent of cases pleural effusion with characteristics of exudate and abundance of lymphocytes and eosinophils may develop. About 25 percent of pleural effusion cases lead to empiema [12]. In more severe manifestations, diffuse pneumonia, fever, and respiratory insufficiency, as well as acute respiratory distress syndrome, may present [13].

In about 1 out of 20 cases, a pulmonary nodule or pulmonary cavity may develop during the infiltrate resolution phase. The differentiation between these morphological changes in lungs and malignancy is challenging even by using positron emission tomography. In such cases, precise collection of the anamnesis can be very helpful: young age (<55), absence of past lung diseases, a job in the farming or construction sector, or traveling in endemic regions will suggest further examination for coccidioidomycosis. To confirm the diagnosis, microbiological culture, histopathological bioptate analysis, or serological testing should be used [14,15].

Diagnosis of coccidioidomycosis can be proven by taking a culture from any clinical site (e.g., bronchoalveolar lavage or cerebrospinal fluid). Colonies of *C. immitis* usually take from 3 to 5 days to grow, but if the patient receives antifungal therapy during the collection of the specimen, colonies may grow up to 3 weeks [15,16]. If bioptate is taken, spherical structures with endospores are usually seen while examining it. Contrary to arthroconidia, the spherules size ranges from 60 to 100 μm. Fungal hyphae and other morphological fungal structures in cases of this infection are rarely seen [17,18].

In endemic regions, one of the most widespread ways of confirming the diagnosis is serological testing combined with a clinical evaluation. It is usually conducted by detecting specific G and M class immunoglobulins using the immunodiffusion method. For diagnosis confirmation in immunocompetent patients and the surveillance of disease progress, the immunoglobulin G complement fixation test may be useful. Complement fixation titers above 1:16 could indicate a higher risk of dissemination. It is necessary to emphasize that immunosuppressed patients’ serological testing can be false negative in about 20–50 percent of cases [19].

A polymerase chain reaction is not applied in routine clinical practice as the research and refinement of primers for *C. immitis* is still being conducted [20]. Skin tests to evaluate acquired immunity after the disease are also being developed [21].

Even when the diagnosis is made and the treatment is applied, for some patients an extrapulmonary disseminated form of coccidioidomycosis can develop. Some of these patients require surgical intervention when abscesses form or focal/neurological pathophysiological phenomena persist. According to the literature, coccidioidomycosis dissemination is possible not only regarding bones, joints, or skin but also concerning endocrine glands, peritoneum, liver, pancreas, pericardium, bone marrow, kidneys, bladder, or genitals [6,22,23].

The most severe form of coccidioidomycosis is dissemination to the brain and meningitis. This form should be suspected when a patient with a confirmed diagnosis develops central nervous system-related symptoms: headache, photophobia, blurred vision, cognitive dysfunction, and changes in the auditory system. In such cases, lumbar puncture and culture cultivation from the cerebrospinal fluid is needed. The laboratory analysis of cerebrospinal fluid usually shows cytosis, elevated protein and low glucose concentrations [24]. The most common complications of coccidioidomycosis meningitis are hydrocephaly, brain vessel vasculitis, ischemia, vasospasm, or hemorrhage [25].

### 3.2. Treatment

When the diagnosis of coccidioidomycosis is made, the most popular treatment option is the triazole class drug fluconazole. The standard dosage of the drug is 400 mg a day. Its advantages are high bioavailability even when administered perorally and a relatively slow excretion (half-life ranges from 24 to 30 h). It is well tolerated and has good renal clearance [26]. However, hepatotoxicity of fluconazole remains the point of discussion. In the Systematic Review of Antifungal-Induced Acute Liver Failure, Gadour and Kotb note that fluconazole is considered relatively safe and is associated only with minor changes in liver function tests that usually do not require interruption of treatment. Nevertheless, the authors mention that there are described cases when the administration of fluconazole had to be stopped due to liver toxicity [27]. Gayam V. et al. described a case in which administration of intravenous fluconazole resulted in elevated liver enzymes (GOT 25,000 IU/L and GPT 6500 IU/L). Still, it should be mentioned that the patient had comorbidities, such as hypertension, diabetes mellitus, and dyslipidemia, and his status improved after discontinuation of the drug [28]. The drug manufacturer provides a warning that fluconazole should be administered carefully to patients with liver dysfunction [29]. Even though there are no precise therapeutic drug monitoring guidelines for fluconazole, it should be noted that hepatic injury typically arises within the first few weeks of treatment. Recovery, when the drug is terminated, can take from several weeks to 3 months. Therefore, liver function should be monitored during the entire period [30]. 

Fluconazole may also be used to treat coccidioidomycosis related meningitis. In such cases, the therapeutic dosage of the drug is increased to 800–1200 mg a day. For treatment of the most severe forms, a dosage of 2000 mg a day might be prescribed [31]. However, the biggest disadvantage of the drug is that it is incapable of eradicating the fungus. Therefore, it is used only to suppress the disease and must be administered for an unspecified period [26].

If the disease does not respond to treatment with fluconazole, administration of other triazoles (voriconazole, posaconazole or isavuconazole) should be considered. These triazoles demonstrate similar pharmacokinetic and pharmacodynamic properties in vivo and can be administered in analogical doses perorally [32,33]. However, the interaction between other medications that the patient is taking must be considered, while voriconazole blocks the activity of CYP2C9, CYP2C19, and CYP3A4 enzymes. Additionally, voriconazole is thought to be more related to neurotoxic and hepatotoxic side effects than fluconazole [34]. Posaconazole frequently provokes gastroenteric side effects, skin rashes, and elevation of transaminases in blood serum. Also, it should not be forgotten that patients may refuse to use posaconazole because of the high price [35]. Isavuconazoe is a promising drug, but so far shows beneficial effects only in treating coccidioidomycosis pneumonia [32].

If there is no effect of treatment with triazoles, amphotericin B is recommended by the Infectious Diseases Society of America. Transition to intravenous usage of the drug is indicated for patients after two or more surgeries to control the infection who develop severe manifestation of the bone coccidioidomycosis or who develop rapidly progressing disease while being immunocompromised. When coccidioidomycosis manifests as CNS infection, intrathecal usage of the drug is suggested. It must be noted that in most cases, triazoles should be used first, and if the treatment with amphotericin B is started, the dosage should be gradually increased and then decreased if the infection remits [36].

Other drugs that have the potential to suppress or eradicate coccidioidomycosis are currently being investigated. The newest not yet approved drug for the treatment of the disease is Nikkomycin Z. It showed efficacy in clinical trials with rodents, in some cases even eradicating the fungus. However, further research is needed [37].

## 4. Conclusions

Pulmonary coccidioidomycosis is a fungal infectious disease easily diagnosed in endemic regions based on typical radiological, histopathological, and microbiological findings. Nevertheless, the risk of imported cases is constantly rising as traveling rates increase. These cases may be a diagnostical challenge because of their rareness in non-endemic parts of the world. As seen from the clinical case, coccidioidomycosis should be suspected when there is a history of traveling to an endemic region (Tulare County, California in this case) along with the presentation of respiratory symptoms, eosinophilia, and persisting fever. Conventional treatment of the infection includes the administration of triazoles or amphotericin B if the latter fails. However, it must be stressed that total eradication of the fungus is hardly possible, and patients should be constantly monitored. 

## Data Availability

The data presented in this case report are available on request from the corresponding author.
